# Acute Kidney Injury in Neonates: From Urine Output to New Biomarkers

**DOI:** 10.1155/2014/601568

**Published:** 2014-03-05

**Authors:** Alexandre Braga Libório, Klébia Magalhães Pereira Castello Branco, Candice Torres de Melo Bezerra

**Affiliations:** ^1^Public Health Postgraduate Program, Universidade de Fortaleza, UNIFOR, Fortaleza, CE, Brazil; ^2^Internal Medicine Department, Faculdade de Medicina, Universidade Federal do Ceará, Avenida Abolição No. 4043, Ap. 1203 Edifício Jangada, Mucuripe, 60165-082 Fortaleza, CE, Brazil

## Abstract

In the past 10 years, great effort has been made to define and classify a common syndrome previously known as acute renal failure and now renamed “acute kidney injury (AKI).” Initially suggested and validated in adult populations, AKI classification was adapted to the pediatric population and recently has been modified for the neonatal population. Several studies have been performed in adults and older children using this consensus definition, leading to improvement in the knowledge of AKI incidence and epidemiology. In spite of these advances, the peculiar renal pathophysiology of critically ill newborn patients makes it difficult to interpret urine output (UO) and serum creatinine (SCr) levels in these patients to diagnose AKI. Also, new urine biomarkers have emerged as a possible alternative to diagnose early AKI in the neonatal population. In this review, we describe recent advances in neonatal AKI epidemiology, discuss difficulties in diagnosing AKI in newborns, and show recent advances in new AKI biomarkers and possible long-term consequences after AKI episode.

## 1. Introduction

Acute kidney injury (AKI), previously known as acute renal failure, is defined by an acute and reversible increment in serum creatinine (SCr) levels associated or not with a reduction in urine output (UO) oliguria/anuria. It is a complex disorder with clinical manifestations ranging from mild injury to complete kidney failure, generally requiring renal replacement therapy, peritoneal dialysis or hemodialysis. Evidence shows that it is not just a marker of illness severity in the pediatric or adult patients but that it has a direct association with poor outcomes [1, 2].

The epidemiology of AKI has changed in the past few decades, with rapid advances in medical technology. Recent studies recognize that even small increments in serum creatinine (SCr) levels increase morbidity and mortality [3, 4]. Moreover, it has been clearly demonstrated that AKI has implications in long-term outcomes in adults and children, compromising survival and increasing chronic kidney disease incidence [5].

The prevalence of in-hospital AKI is high and the incidence of AKI secondary to another systemic illness or another treatment is now higher than that of primary renal disease [6]. Due to different definitions, it was difficult to determine AKI real incidence, especially in children. Recently, new definition and classification [7] have renewed the interest in this topic and new studies in older children and neonatal population are being performed. Furthermore, the advances in new biomarkers that enhance clinical diagnosis capacity and the implementation of possible early treatment have promoted the study of AKI in children. Despite these advances and new technologies in renal replacement therapies, the mortality rate from AKI remains elevated and, at least in the adult population, has remained stable over the past 50 years [8].

In newborns patients, AKI importance and dilemmas are even more pronounced, as a newborn's kidneys are more susceptible to hypoperfusion and have low glomerular filtration rate, high renal vascular resistance, high plasma renin activity, decreased intercortical perfusion, and decreased reabsorption of sodium in the proximal tubules. All these features make newborns more susceptible to injury in the first days of life. Moreover, difficulties in SCr interpretation (discussed below) make it more difficult to achieve a consensus regarding AKI definition. Because of all these difficulties in diagnosing AKI in newborns, the new biomarkers are expected to be of greater importance in AKI approach in high-risk neonatal populations.

## 2. Acute Kidney Injury Definition

Until recently, AKI definition was broad, which made it difficult to establish comparisons between different studies. Different definitions can be responsible for the difficulties in discerning its real epidemiology and prognosis implications. In 2004, the Acute Dialysis Quality Initiative (ADQI) proposed an AKI classification system called “Risk, Injury, Failure, Loss, End-Stage Kidney Disease (RIFLE)” criteria to promote a consistent and consensus AKI definition [9]. The RIFLE criteria remained to be based on routinely used parameters: acute changes in serum creatinine and reduced urine output (UO)—Table 1. Many studies in different populations, comprising more than 500,000 patients, have validated these criteria in adult populations, correlating AKI severity with early and late mortality, length of hospital stay, and other outcomes [10].

Three years later, RIFLE criteria were modified to be used in children, thus giving rise to the pediatric RIFLE criteria or pRIFLE [7]. The authors adapted glomerular filtration rate (GFR) decline criteria from adults and maintained the same urine output (UO) definition—see Table 1. The main difference is a lower cutoff SCr to achieve the F category, requiring a GFR lower than 35 mL/min instead of a SCr of 4.0 mg/dL. Several studies have also validated pRIFLE in children, mainly in critically ill patients or after cardiac surgery [11–16]. After that, it was mathematically demonstrated that it is not necessary to calculate the GFR and that, by using only the SCr increment, it was possible to diagnose and to assess AKI severity [17]. Also, the Acute Kidney Injury Network (AKIN) revised AKI classification and has adopted the percentage SCr increment to diagnose AKI, recognizing that eGFR was not necessary. This is important because it made it possible to diagnose AKI in retrospective studies, in which the researchers often do not have access to children's length, which is necessary to calculate eGFR. Both the AKIN and pRIFLE definitions have been used in several pediatric studies and have been shown to be similar when compared in critically ill and noncritically ill children [17].

Few studies have evaluated pRIFLE or the similar AKIN criteria [18] specifically in neonates [19, 20] and none has applied UO criterion in this population. Considering major differences in the neonatal population, mainly in preterm infants (immature tubular cells, higher total body water, and maternal SCr influence), it is difficult to accept all pRIFLE definitions for the neonatal population. A recent study by our group [21] proposed the evaluation of urine output and its impact on outcomes in a critically ill neonatal population. Urine output cutoffs higher than 1.5 mL/Kg/24 h were associated with higher mortality, mechanical ventilation duration, and length of hospital stay. These urine output criteria were incorporated to pRIFLE and named neonatal RIFLE, or nRIFLE [22]. Table 1 discloses a comparison between adult, pediatric, and neonatal RIFLE.

Although some studies have recently applied pRIFLE criteria in neonatal populations, mainly in those undergoing cardiac surgery, no other study has included UO criterion and this is a subject that needs further validation. Using UO as a biomarker of neonatal AKI can be especially important due to difficulties in evaluating SCr at this population, as explained below. Finally, the importance of these definitions is highlighted by the need to clarify AKI epidemiology, direct our research efforts, and ultimately improve prognosis.

## 3. Epidemiology 

While many studies have emerged in recent years on AKI epidemiology in older children, only a few have evaluated its incidence and clinical importance in newborn patients. Moreover, recent studies with modern AKI definition included only special neonatal populations: postneonatal asphyxia [20], low birth weight [23], and after cardiac surgery [24]. Data on AKI epidemiology in a general neonatal ICU has been scarcely studied [21].

Critically ill neonates are at greater risk of having AKI as they are commonly exposed to nephrotoxic medications and have frequent infections, which leads to multiorgan failure [25]. Older studies estimated that AKI incidence in this population was between 8 and 24% [26] and a recent study, using UO criterion in addition to SCr, disclosed an incidence of almost 20% [21]. The importance of different definitions of AKI is highlighted by comparison with another new study that used only SCr, where AKI was identified in only 6.3% of newborn patients [27].

Another neonatal population at especially high risk of having AKI is that with postneonatal asphyxia. The incidence of asphyxia is estimated at between 1 and 10 per 1,000 live births and these patients are prone to multiorgan dysfunction and a redistribution of cardiac output to maintain cerebral, cardiac, and adrenal perfusion, while potentially leading to renal ischemia. Because of the different definitions, AKI incidence postneonatal asphyxia is reported in up to 30% to 56% of cases [28–31]. Newborns with AKI after postneonatal asphyxia have an ominous prognosis, particularly those with oliguric AKI [32].

AKI incidence in newborns with congenital heart disease undergoing cardiopulmonary bypass has been assessed in several studies. In a recent prospective multicenter study using pRIFLE criteria [19], AKI occurred in 64% of the neonates, with 55% in stage 1, 20% in stage 2, and 25% in stage 3. Hospital stay, ICU stay, and mechanical ventilation were longer in those newborns with AKI. Also, in-hospital mortality rate was greater, particularly in those with AKI stage 3 and those requiring dialysis. Interestingly, newborns with advanced AKI (stages 2 or 3) had lower height after a two-year follow-up even after adjustment to other variables, demonstrating that AKI has long-term implications, as discussed below.

Two recent studies investigated AKI incidence in low birth weight newborns. Koralkar et al. [23] performed a study in very low birth weight infants (<1.500 g) and demonstrated that 18% of the VLBW infants developed AKI and these had a mortality rate 2.4 times greater than the others. In one case-control study in VLBW infants [33], high mean airway pressure, low blood pressure, and the use of cefotaxime were associated with AKI. Finally, in a multicenter study, neonates on extracorporeal membrane oxygenation (ECMO) had an AKI incidence greater than 80% and AKI was associated with an adjusted mortality rate that was 3.2 times higher [34].

## 4. Difficulties to Define Acute Kidney Injury in Neonatal Period

Despite these functional classification systems, the diagnosis of AKI is troublesome, as current diagnosis is based on two main findings: changes in SCr and urine output. As in the adult population, both are late effects of injury and not markers of the injury itself and, moreover, neonatal physiology complicates the interpretation of these biological markers [35].

Some important points need to be considered with any neonatal AKI definition and diagnosis. It is generally believed that neonates have nonoliguric AKI [26], but this can be a misconception due to the lack of knowledge about normal UO in critically ill newborns. The total body water content is greater in newborns than in adult patients. Especially in preterm infants, the total body water can be as high as 80% of body weight [36]. This difference in water content, in addition to immature tubular development, can explain why UO in newborns is normally greater than in other populations. So, it was demonstrated that urine output less than 0.5 mL/kg per hour is an nonsensitive marker of AKI and this level must be increased to up to 1.5 mL/Kg per hour [21]. This urine output level can be even higher in preterm newborns, because of immature tubular development. In our study, we identified that diuresis as high as 1.5–2.0 mL/kg/h is associated with a fatal outcome in LBW infants.

Serum creatinine is the most practical and often used method to monitor glomerular filtration rate, but its use in the neonatal period is associated with some limitations. During the first 48–72 h of life, neonatal SCr still reflects maternal levels and these values may decline at varying rates over days, depending on gestational age [26]. Thereby, the levels of SCr during the first week after birth and its changes (or lack of change) may be difficult to interpret. Moreover, SCr concentrations may not change until 25–50% of the kidney function has already been lost and, at lower GFR, SCr will overestimate renal function due to tubular secretion of creatinine.

Other additional factors need to be recalled: normal nephrogenesis begins at 8 weeks of gestation and continues until the 34th week. Thereafter, GFR improves steadily over the first few months of life. Depending on the degree of the neonate's prematurity, GFR steadily improves during the first week of life, concomitantly with alterations in renal blood flow. Overall GFR in term and preterm infants is very low, and there is a very wide distribution of normal SCr values, which vary greatly, depending on the level of prematurity and age [31].

## 5. New Biomarkers in Neonatal Acute Kidney Injury

Because the incidence of AKI is still high and the outcome remains poor, research has focused on identifying new biomarkers able to anticipate the AKI diagnosis in hours or even days before a UO reduction or SCr increment can be detected. Early AKI diagnosis would bring out new perspectives in therapeutic possibilities. Moreover, the knowledge of novel serum and urine biomarkers may change the approach to AKI and can help to differentiate between different causes of established AKI and implementing preventive interventions. Studies on AKI biomarkers in neonates are limited and mainly performed in specific populations at risk for AKI, such as VLBW, asphyxiated newborns, and children who undergo cardiac surgery with cardiopulmonary bypass (CPB). Studies on biomarkers predicting AKI in general in critically ill neonates are lacking.

There are many challenges in validating AKI biomarkers. Large-scale observational multicenter studies in different critically ill populations and healthy neonates are needed to characterize the differences between the course of SCr and biomarkers during the length of AKI. Also, validation of new biomarkers in clinical practice and testing of markers as predictive factors for endpoints, such as LOS, needed for renal replacement therapy, mechanical ventilation time, and mortality are necessary.

**Figure 1:**
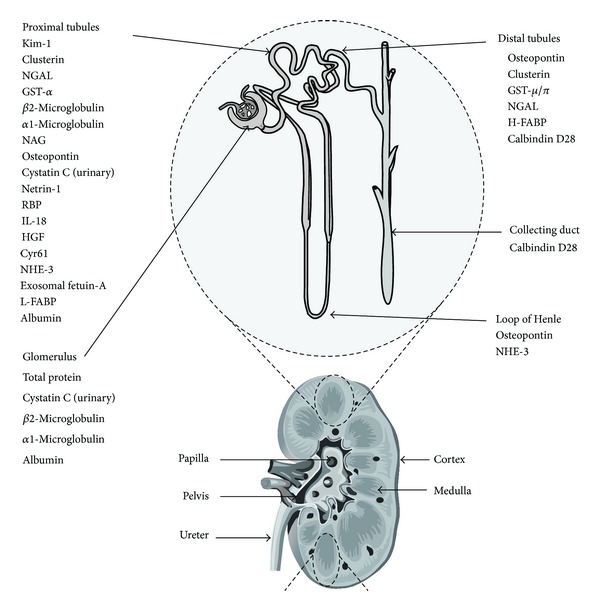
Nephron segment-specific biomarkers of kidney injury. Adapted from [40] with permission.

One of the main difficulties is that the new candidate biomarkers are generally tested against SCr, with all the above mentioned problems when used as a gold marker of AKI. Moreover, just like SCr, several studies have shown that some urinary biomarker concentrations depend on gestational age and birth weight [37, 38]. The inability of immature tubules to reabsorb these proteins is underdeveloped in preterm infants and can lead to different values in this population. In recent studies, the most promising early noninvasive AKI biomarkers were serum cystatin C (CysC), urinary interleukin-18 (IL-18), serum and urinary neutrophil gelatinase-associated lipocalin (N-GAL), kidney molecule-1 (KIM-1), osteopontin (OPN), and beta-2 microglobulin (B2mG)—Table 2 [39]. Another recent and promising biomarker is angiotensinogen [40]. Urinary angiotensinogen and its association with renin have been associated with severe AKI and need of renal replacement therapy [41, 42]; however, studies focusing on neonatal AKI are lacking. Generally, renal biomarkers are abnormally expressed protein in renal injury that can reflect lesion in several nephron sites and do not necessarily reflect a GFR reduction—Figure 1.

Cystatin C (CysC) is a cysteine proteinase inhibitor expressed in all nucleated cells produced at a constant rate and cleared exclusively by the kidney. Apparently, CysC does not cross the placenta [48] and, thus, reflects the renal function of the neonates in early postnatal life regardless of body composition and size [49]. Serum CysC (sCysC) was measured in 62 preterm neonates with respiratory distress syndrome (RDS) and 34 control neonates without RDS and sCysC was identified as an earlier marker of AKI in preterm neonates with RDS, increasing before SCr [47]. The urinary CysC (uCysC) was also predictive of AKI, in nonseptic critically ill newborns [45]. In another small study, enrolling only 13 asphyxiated newborns, uCysC was a sensitive and early AKI biomarker. Although other studies have evaluated CystC in older children, it is difficult to extend their results to the neonatal population, considering that normal values of uCysC decrease with tubular maturation.

Interleukin-18 (IL-18) is a proinflammatory cytokine that is cleaved by caspase-1 and is released in the proximal tubule after AKI [50]. Li et al. [45] enrolled 62 nonseptic critically ill neonates and urine was collected every 48–72 h during the first 10 days of life. They demonstrated that both urinary concentration of CysC (cited above) and IL-18 were associated with AKI, even after controlling for gestational and postnatal age, birth weight, gender, Apgar score, and neonatal acute physiology in nonseptic critically ill neonates. Apparently, uIL-18 has a potential advantage of not changing its normal value with increasing renal maturity but can be influenced by sepsis, reducing its ability to detect AKI.

In the only study evaluating OPN in neonatal population [43], a nested case-control study with 30 patients (only 9 with AKI) showed that AKI newborns had greater OPN values than controls. Prospective studies are lacking to ascertain OPN value in predicting neonatal AKI. Furthermore, we could not identify any study evaluating the role of KIM-1 specifically in a neonatal population.

Urinary NGAL appears to be the most promising AKI biomarker and it is the most strikingly upregulated gene and overexpressed protein in the kidney after ischemia [51]. Many studies have evaluated urinary and serum NGAL capacity to predict AKI in both adults [52, 53] and older children [54, 55]. Specifically in the neonatal population, serum and urine NGAL were evaluated in patients after CPB surgery [44]. Plasma and serum NGAL collected 2 hours after CPB were able to predict AKI with a sensitivity and specificity of nearly 90%. Another study evaluated uNGAL at day 1 in preterm infants and after full adjustment for other factors; it remained significantly associated with AKI development [56].

Another approach to improve AKI diagnosis is to combine multiple biomarkers. Askenazi et al. [43] evaluated 8 candidates for urinary biomarkers: NGAL, IL-18, KIM-1, OPN, B2mG, and CysC. They explored the individual and combined abilities of these biomarkers to predict AKI and mortality in VLBW infants and showed that the combination of three biomarkers was not better than their isolated use; however, this study was only a nested case-control type. Improving our ability to diagnose AKI, maybe with the concomitant use of multiple markers, will allow the implementation of more effective preventive and therapeutic measures to improve AKI prognosis. Finally, some studies have included newborns and older children when evaluating AKI biomarkers [57]. This can lead to erroneous interpretations, as cutoff values can be different even in those patients with different gestational ages and birth weights.

## 6. Long-Term Consequences of Neonatal AKI

Acute kidney injury has been long thought of as a completely reversible syndrome. However, over the past several years, a plethora of data from experimental animal and human studies have been published, indicating that AKI more than likely results in permanent kidney damage (i.e., chronic kidney disease—CKD) and may also result in damage to nonrenal organs [58–60]. Moreover, mortality is higher in adults surviving an AKI episode [61].

In children, it is already known that there is a considerable risk of long-term renal sequelae associated with AKI caused by intrinsic kidney disease, such as Henoch-Schonlein purpura or hemolytic uremic syndrome [62, 63]. However, long-term renal prognosis of AKI in children without primary renal disease was only recently studied. In a prospective cohort study, 126 children were followed for up to three years after AKI episode [5]. Overall, 10% of patients developed CKD and the prevalence had a direct correlation with AKI severity, affecting 17% of patients with AKI stage 3. In this cohort, only 30 patients had neonatal AKI and 5 (16.6%) developed CKD, suggesting that this population is of higher risk at developing CKD after AKI episode. In the only study, evaluating AKI long-term sequelae in newborn population, height was reduced in those with AKI after a two-year follow-up [19]. Studies evaluating CKD after AKI episode, specifically in newborn patients, are lacking.

## 7. Conclusions

Acute kidney injury is a syndrome with severe early and long-term consequences. Recent advances in definition and classifications may improve epidemiologic and clinical studies in the pediatric population, including newborns. New biomarkers are important for an early diagnosis and can have a prominent importance in neonatal population, in which UO can be increased by immature tubules and SCr can suffer maternal influence.

## Figures and Tables

**Table 1:** Comparison between AKI classification system in adults, older children, and newborns.

	Creatinine criteria		Urine output criteria		
	RIFLE	pRIFLE and nRIFLE	RIFLE	pRIFLE	nRIFLE
Risk	Increased creatinine x1.5 or GFR decreases >25%	Increased creatinine x1.5 or GFR decreases >25%	UO ≤0.5 mL/kg/h × 6 h	UO ≤0.5 mL/kg/h × 8 h	UO <1.5 mL/kg/h for 24 h

Injury	Increased creatinine x2 or GFR decreases >50%	Increased creatinine x2 or GFR decreases >50%	UO ≤0.5 mL/kg/h × 12 h	UO ≤0.5 mL/kg/h × 16 h	UO <1.0 mL/kg/h for 24 h

Failure	Increased creatinine x3 or GFR decreases >75% or creatinine >4 mg/dL (acute rise of >4 mg/dL)	Increased creatinine x3 or GFR decreases >75% or GFR <35 mL/min/1.73 m^2^	UO ≤0.3 mL/kg/h × 24 h or anuria × 12 h	UO ≤0.3 mL/kg/h × 24 h or anuria × 12 h	UO <0.7 mL/kg/h for 24 h or anuria for 12 h

**Table 2:** Biomarkers in neonatal acute kidney injury.

Author	Study design	AKI definition	Population	Biomarkers	Main findings
Askenazi et al. [43]	Nested case-control	AKIN	Very low birth weight infants (*n* = 30, AKI = 9)	Urine NGAL and OPN	Urine biomarkers were higher in those with AKI. No information about early AKI diagnosis.

Krawczeski et al. [44]	Prospective cohort	AKIN	374 infants (35 neonates, AKI = 8) undergoing cardiac surgery with CPB	Serum and urine NGAL	Serum and urine NGAL 2 h after CPB are early predictive biomarkers for AKI.

Askenazi et al. [37]	Nested case-control	SCr ≥1.7 mg/dL >72 h after birth or rising values >0.3 mg/dL within 48 h (AKIN)	Neonates birth weight > 2000 g, GA >34 weeks, 5-minute score Apgar ≤7 (*n* = 58, 9 neonates developed AKI)	Urine NGAL, OPN, uCysc, albumin, *β*2 microglobulin, epithelial growth factor, UMOD, and KIM-1	Urine CysC, UMOD, and epithelial growth factor were higher in those with AKI. No information about early AKI diagnosis.

Li et al. [45]	Prospective cohort	SCr >1.5 mg/dL or pRIFLE	Nonseptic critically ill neonates (*n* = 62, AKI = 11)	uCysC and uIL-18	Urine CysC and IL-18 are predictive of AKI in nonseptic critically ill neonates.

Sarafidis et al. [46]	Prospective cohort	SCr ≥1.5 mg/dL >24 h or rising values >0.3 mg/dL from DOL 1	13 asphyxiated neonates and 22 nonasphyxiated (*n* = 35, AKI = 8)	Serum CysC and NGAL Urine CysC, NGAL, and KIM -1	Serum NGAL and uNGAL and uCysC are higher in asphyxiated neonates, even in those not developing AKI. They had also provided an early AKI diagnosis.

Elmas et al. [47]	Case-control	pRIFLE or SCr ≥1.5 mg/dL on the first 3 days	Preterm neonates with RDS (*n* = 28, AKI = 8). Additional control group with 34 neonates without RDS nor AKI	Serum CysC	Serum CysC is an independent predictor of AKI in RDS neonates.

CPB: cardiopulmonary bypass; RDS: respiratory discomfort syndrome; CysC: serum cystatin C; sNGAL: serum neutrophil gelatinase-associated lipocalin; uCysC: urine CysC; uNGal: urine NGAL; KIM-1: kidney injury molecule-1; OPN: osteopontin; B2mG: beta-2 microglobulin; IL-18: interleukin-18; DOL 1: day of life; GA: gestational age; UMOD: uromodulin; PA: postmenstrual age; CPB: cardiopulmonary bypass; RDS: respiratory distress syndrome.
